# Illegitimate tasks, knowledge hiding, and performance erosion: a four-wave latent change score investigation

**DOI:** 10.3389/fpsyg.2025.1730215

**Published:** 2026-01-23

**Authors:** Honghong Zhu, Xuan Gu, Daokui Jiang, Yaru Liu

**Affiliations:** Business School, Shandong Normal University, Jinan, China

**Keywords:** challenge-hindrance theory, knowledge sharing knowledge hiding, latent change score modeling, performance, task, unnecessary tasks, unreasonable tasks

## Abstract

This four-wave longitudinal study reframes unnecessary and unreasonable tasks as relational stimuli. Latent change score modeling on 250 employees (1 month intervals) showed that within-person increases in unnecessary tasks heightened knowledge hiding (β = 0.30-0.32), which subsequently eroded task performance (indirect effect = −0.12 to−0.14). Unreasonable tasks exerted comparable indirect harm (indirect = −0.11 to−0.12) but carried no significant direct effect on performance (β = −0.08, *p* = 0.183). Thus, both forms of illegitimate tasks damage later performance solely through the amplification of knowledge hiding, challenging static “stressor–strain” assumptions and underscoring the need to curb defective task design before collaborative exchanges are undermined.

## Introduction

Organizations continue to grapple with the persistent problem of “bad jobs” that expose employees to work characteristics widely regarded as detrimental—namely, tasks that are redundant, illogical, or impossible to execute properly (Oldham and Hackman, 2010). Two under-studied yet increasingly salient facets are unnecessary tasks (i.e., redundant or meaningless work that serves no value-added purpose) and unreasonable tasks (i.e., assignments that violate logic, feasibility, or ethical standards). While cross-sectional studies typically treat such demands as classic hindrance stressors that erode motivation and performance (Cavanaugh et al., 2000), employees in modern knowledge contexts often cannot simply withdraw; instead, they must find real-time solutions to complete the work (Tan et al., 2024). Understanding how these problematic demands unfold over time and whether they can spark collaborative coping behaviors is therefore critical for both theory and practice.

Extant research on hindrance demands has relied heavily on between-person, static designs that compare individuals high vs. low on perceived hindrances (Podsakoff et al., 2007). Consequently, we know little about within-person dynamics: do rises in unnecessary or unreasonable tasks prompt employees to share knowledge more frequently, and does such collaboration ultimately sustain task performance? A handful of qualitative studies hint that bureaucratic burdens may unintentionally foster mutual assistance (Grant and Ashford, 2008), but longitudinal, quantitative evidence is absent. Moreover, the challenge-hindrance appraisal framework has recently been re-conceptualized as a dynamic process wherein individuals reappraise initially obstructive demands as challenges or as solvable via collective action (Rodell and Judge, 2009). Yet this theoretical pivot awaits empirical tests capable of modeling intra-individual change while separating initial levels from growth trajectories.

Additionally, prior studies have often conflated different types of hindrance demands, overlooking their dimension-specific effects (Webster et al., 2010). For example, unnecessary tasks may be perceived as more controllable than unreasonable tasks, leading to different coping strategies (Lazarus and Folkman, 1984). Furthermore, knowledge hiding has been identified as a key behavioral mechanism through which employees mitigate work stressors (Wang and Noe, 2010), yet its longitudinal role in response to task design flaws remains underexplored. Finally, most job design research has focused on static outcomes such as job satisfaction or burnout, rather than dynamic performance trajectories (Parker et al., 2017).

To address these gaps, we collected four waves of survey data from 250 knowledge workers at 3 month intervals and applied bivariate latent change score modeling (LCSM) (McArdle, 2009). LCSM decomposes observed scores into an initial level and a latent change factor for each construct, thereby offering a rigorous test of how within-person increases in unnecessary and unreasonable tasks predict corresponding changes in knowledge hiding and subsequent task performance (Kruk et al., 2023). Grounded in challenge-hindrance appraisal theory, we argue that employees who experience growing unnecessary work will appraise it as a controllable hindrance and engage in knowledge hiding as a proactive workaround; in contrast, escalating unreasonable tasks—perceived as less controllable—will evoke direct, effortful problem solving rather than collaborative exchange. We further integrate Conservation of Resources (COR) theory (Hobfoll, 1989) to posit that employees invest in knowledge hiding as a resource-generating strategy to prevent further loss of time and energy. Meanwhile, social functionalism (Durkheim and Coser, 1998) suggests that institutional deficiencies (e.g., poor task design) activate informal adversity-driven defensive withdrawal to maintain system functionality. Thus, we expect different temporal pathways for unnecessary vs. unreasonable tasks.

**Figure 1:**
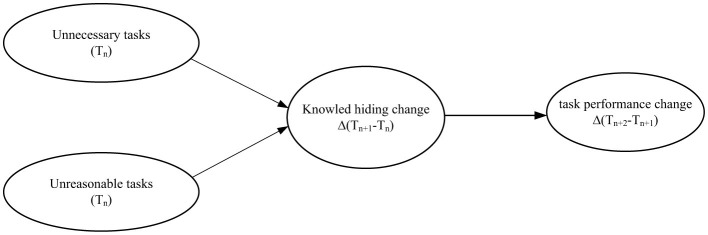
Research model.

This study addresses the following question: Do within-person increases in illegitimate tasks (unnecessary vs. unreasonable) trigger knowledge hiding, and does this behavioral response subsequently erode task performance over time? We propose that both task types, by violating implicit contracts about meaningful work, will activate resource-protection motives leading to knowledge concealment. Figure 1 illustrates our latent change score model, which disaggregates between-person and within-person variance to test temporal mediation. By modeling intra-individual change across 4 monthly waves, we provide a dynamic test of how “bad tasks” become performance liabilities through the micro-mechanism of knowledge hiding.

The present study makes four primary contributions. First, by modeling intra-individual change rather than between-person differences, we provide the first longitudinal evidence that “bad tasks” can generate functional, collaborative behaviors over time, refining the predominantly static challenge-hindrance literature (Rodell and Judge, 2009). Second, we unpack the dimension-specific effects of unnecessary vs. unreasonable tasks, demonstrating that only the former stimulates knowledge hiding, whereas the latter lifts performance through a direct, non-collaborative pathway. Third, our use of LCSM advances methodology in job design research by explicitly separating baseline levels from dynamic slopes, thereby offering stronger causal inference regarding the temporal ordering of demands, sharing behaviors, and performance outcomes (McArdle, 2009). Finally, we integrate COR theory and social functionalism to explain why employees convert task adversity into collective gain, offering a multi-level theoretical account of “adversity-driven collaboration” in contemporary organizations.

## Theory and hypotheses

### Theoretical foundation

Recent advances (2020–2024) have refined our understanding of illegitimate tasks and knowledge dynamics. Meta-analytic evidence (Pindek et al., 2021) confirms illegitimate tasks as robust predictors of counterproductive work behaviors (Yuan and Yan, 2024), while daily diary studies (Gabriel et al., 2023) reveal their dynamic spillover effects. Regarding knowledge hiding, Grošelj et al. (2020) meta-analysis (*k* = 65, *N* = 21,000) identifies perceived organizational injustice as a primary antecedent, with stronger effects in high power-distance cultures (Jiang et al., 2023). Critically, Katz et al. (2023) reconceptualize illegitimate tasks as “stress-as-offense-to-self,” emphasizing resource threat over challenge appraisal. These dynamic perspectives highlight a crucial gap: no longitudinal study has modeled within-person change processes linking illegitimate tasks, knowledge hiding, and performance trajectories using latent change score modeling (LCSM), which offers stronger causal inference than cross-lagged panels (Hamaker et al., 2015; Kottwitz et al., 2025).

This study integrates challenge-hindrance appraisal theory (Cavanaugh et al., 2000; Rodell and Judge, 2009) with Conservation of Resources (COR) theory (Hobfoll, 1989) and social functionalism (Durkheim and Coser, 1998) to explain why within-person increases in two forms of “bad” tasks—unnecessary vs. unreasonable—activate distinct behavioral pathways that ultimately influence task performance. Challenge-hindrance theory posits that stressful demands are interpreted as either controllable hindrances or uncontrollable threats; controllable hindrances stimulate proactive, collaborative coping, whereas uncontrollable hindrances elicit direct or solitary effort (Rodell and Judge, 2009). COR theory adds that employees invest existing resources (e.g., expertise) to generate new resources (e.g., social support) when threatened (Hobfoll, 1989). Social functionalism further contends that institutional deficiencies (e.g., poor task design) trigger informal adversity-driven defensive withdrawal to maintain system functionality (Durkheim and Coser, 1998). Synthesizing these perspectives, we propose an asymmetric mediation model in which knowledge hiding change transmits the effect of unnecessary-task change, but not unreasonable-task change, to performance change.

### Unnecessary tasks and knowledge-hiding change

Challenge-hindrance appraisal theory contends that when employees encounter job demands they perceive as controllable, they are inclined to interpret them as surmountable hindrances rather than threats (Cavanaugh et al., 2000; Rodell and Judge, 2009). Unnecessary tasks—work that is redundant, low-value, or bureaucratic—precisely fit this appraisal profile because individuals believe the waste can be reduced through better coordination (Webster et al., 2010). From a conservation-of-resources (COR) perspective, such appraisals trigger proactive investment of existing resources (time, expertise) to generate new resources (social support, reciprocity) that prevent further resource drain (Hobfoll, 1989). Knowledge hiding represents a low-cost, high-leverage investment: exchanging tips, templates, or work-arounds can eliminate duplicated effort and simultaneously build interpersonal resources (Wang and Noe, 2010). Recent experience-sampling studies show that daily fluctuations in redundant work predict same-day elevations in help-seeking and information giving (Koopman et al., 2016), while a three-wave panel found that employees who reported increasing bureaucratic demands subsequently increased collaborative problem-solving (Tims et al., 2015). Because unnecessary tasks are appraised as controllable and remediable, within-person increases should stimulate individuals to pool collective knowledge.

*H1: Within-person increases in unnecessary tasks will be positively related to within-person increases in knowledge hiding*.

### Unreasonable tasks and knowledge hiding change

Unreasonable tasks—assignments that are illogical, ethically dubious, or impossible to complete satisfactorily—are typically appraised as low in controllability and high in self-threat (Lazarus and Folkman, 1984). Challenge-hindrance theory predicts that when hindrances are viewed as uncontrollable, employees shift from collaborative sense-making to direct compliance or private problem-solving in order to avoid blame or reputational damage (Pham Thi and Duong, 2024; Rodell and Judge, 2009). COR theory adds that under high threat and low control, individuals conserve rather than expend resources; sharing proprietary knowledge may be judged too risky if the assignment itself is irrational (Hobfoll, 1989). Longitudinal evidence supports this reasoning: a four-wave study found that rises in illegitimate tasks did not predict subsequent collegial helping but did predict solitary extra-role effort (Eatough et al., 2015). Similarly, an experience-sampling investigation showed that daily unreasonable requests increased employees' silent compliance behaviors while decreasing cooperative voice (Koopman et al., 2016). Consequently, within-person growth in unreasonable tasks should not elicit greater knowledge hiding.

*H2: Within-person increases in unreasonable tasks will NOT be significantly related to within-person increases in knowledge hiding*.

### Knowledge hiding change and task-performance change

Knowledge hiding delivers three performance-relevant resources: diversified solution sets, rapid error correction, and socio-emotional support that sustains self-efficacy (Wang and Noe, 2010). Meta-analytic evidence indicates that individual-level knowledge-hiding behaviors correlate ρ = 0.34 with supervisor-rated task performance (Låstad et al., 2025). Longitudinally, a four-wave study of R&D engineers revealed that within-person increases in daily knowledge hiding predicted objective project output 3 months later (Tims et al., 2015). From a social-functionalism angle, informal knowledge exchange functions as a micro-repair mechanism that compensates for formal system defects (Durkheim and Coser, 1998); when employees pool know-how, they shorten learning curves and reduce redundant iterations, thereby maintaining output quality even under sub-optimal task conditions. Thus, as individuals share more knowledge over time, their own task performance should improve.

*H3: Within-person increases in knowledge hiding will be positively related to within-person increases in task performance*.

### Mediating role of knowledge-hiding change

Building on the logic developed in H1–H3, we argue that knowledge-hiding change functions as a key behavioral conduit through which escalating unnecessary tasks translate into performance gains, whereas unreasonable tasks bypass this collaborative route and exert only a direct effect on performance.

First, unnecessary tasks are appraised as controllable hindrances that can be alleviated through joint sense-making (Rodell and Judge, 2009). When employees experience a within-person uptick in redundant or low-value work, they proactively exchange templates, short-cuts, and coordination scripts to eliminate duplication (Kunzelmann et al., 2025; Wang and Noe, 2010). This incremental knowledge pooling shortens task completion time, reduces error rates, and signals cooperative intent to supervisors—objective and symbolic benefits that feed directly into performance evaluations (Låstad et al., 2025). Longitudinal evidence supports this causal chain: a four-wave panel of R&D engineers revealed that increases in daily knowledge hiding predicted supervisor-rated performance 3 months later (Tims et al., 2015). Thus, knowledge-hiding change should statistically mediate the effect of unnecessary-task change on performance change.

Second, unreasonable tasks are appraised as uncontrollable and self-threatening (Lazarus and Folkman, 1984). Employees respond with private, effortful compliance rather than open collaboration, fearing that public questioning may damage credibility (Eatough et al., 2015; Schilbach et al., 2023). Because knowledge-hiding change is not significantly triggered by unreasonable-task change (cf. H2), an indirect effect through this pathway is unlikely. Instead, unreasonable tasks may elevate performance through alternative mechanisms such as impression management or intensive solo problem-solving (Webster et al., 2010).

Taken together, we propose an asymmetric mediation model: knowledge-hiding change transmits the benefits of unnecessary tasks but not of unreasonable tasks to performance.

*H4a: Within-person increases in knowledge hiding will mediate the positive relationship between within-person increases in unnecessary tasks and task-performance change*.

*H4b: The indirect effect of unreasonable-task change on performance change via knowledge-hiding change will be non-significant*.

## Research design

### Participants and procedure

The data collection process can be summarized into the following steps: Firstly, the translation and simplification of the scale. The accuracy and original meaning of the scale are ensured through the translation and back-translation process. At the same time, to reduce the burden on participants and improve the quality of the responses, a simplified version of the scale was used for the knowledge hiding behavior.

Secondly, the research design and selection of research subjects. To facilitate sampling, full-time employees from 18 companies- selected by two-stage stratified random sampling (18 firms drawn from the provincial register followed by 10-30 employees per firm chosen via anonymized rosters)- were randomly selected as the subjects of the survey. Each company conducted the survey through the head of the human resources department, with a sample size ranging from 3 to 30 people, including positions such as marketing, research and development, production, administration, finance, etc. The human resources department obtained the consent of the participants to ensure voluntary participation. To ensure the confidentiality of the survey data, all participants were identified only by a unique ID.

Next, the pre-survey and screening. The pre-survey was conducted on the last working day of the second week in April, and confirmation was made the next day for those who did not respond. Information such as gender, age, education level, work experience, job category, job level, and company type were collected, and a total of 350 people were solicited to fill out the questionnaire.

Next, the data collection time and method. The first formal collection was on the last working day of the third week in May, with a requirement to complete it before Saturday, collecting other questions except for demographic variables, and after matching, a total of 337 valid questionnaires were received; a month later, based on the first valid sample, the second formal collection was on the last working day of the third week in June, with a requirement to complete it before Saturday, collecting other questions except for demographic variables, and after matching, a total of 317 valid questionnaires were received; another month later, based on the second valid sample, the third formal collection was on the last working day of the third week in July, with a requirement to complete it before Saturday, collecting other questions except for demographic variables, and after matching, a total of 251 valid questionnaires were received; for all surveys, matching was done through a unique ID, and it was set that only one questionnaire could be filled per IP.

Finally, reducing sample loss and reward mechanism. Unqualified questionnaires were excluded based on the following criteria: attention test questions, such as selecting Beijing as the capital; short filling time, such as less than 2 mins; random filling, such as all options being the same; whether they participated in the previous round of the survey. To encourage serious and responsible completion, each participant received a cash red envelope reward of 10-15 yuan after completing the questionnaire each time.

### Variables measurement

All the measures were translated from English to Chinese following the translation-back translation procedure by Brislin (1980).

Illegitimate Task. Illegitimate tasks consist of two dimensions: unnecessary tasks and unreasonable tasks. These are measured using an 8-item scale developed by (Semmer et al., 2010), where participants are asked to make choices on a 5-point scale (ranging from 1 for “strongly disagree” to 5 for “strongly agree”). Unnecessary tasks include four items, such as “I have to deal with tasks that are completely unnecessary” and “I have to deal with tasks that are entirely meaningless.” The Cronbach's alpha for these four measurements was 0.89, 0.90, 0.88, and 0.89, respectively; unreasonable tasks also include four items, such as “I have to deal with tasks that should be done by someone else” and “I have to deal with tasks that are irrelevant to me and should not be my responsibility.” The Cronbach's alpha for these four measurements was 0.91, 0.92, 0.93, and 0.94, respectively.

Knowledge Hiding Behavior. Knowledge hiding behavior is measured using a modified scale developed by (Rhee and Choi, 2016), where leaders are asked to rate their subordinates' behavior on a 5-point scale (ranging from 1 for “completely non-compliant” to 5 for “fully compliant,”) including four items, such as “Promises to inform the subordinate, but actually has no intention of doing so” and “Even though the subordinate knows this information, they might claim not to know.” The Cronbach's alpha for these four measurements was 0.91, 0.92, 0.90, and 0.91, respectively.

Task Performance. Task performance is measured using a modified scale developed by (Van Dyne and LePine, 1998), where leaders are asked to rate their subordinates' performance on a 5-point scale (ranging from 1 for “completely non-compliant” to 5 for “fully compliant”), including four items, such as “Fully completes the assigned work tasks” and “Work performance meets job performance standards.” The Cronbach's alpha for these four measurements was 0.96, 0.95, 0.94, and 0.94, respectively.

### Analytic strategies

We specified a bivariate latent change score model (McArdle, 2009) in Mplus 8.8 following four analytic steps. Power analysis using Monte Carlo simulation (Mplus) indicated that *N* = 250 provides 0.89 power to detect the observed indirect effect (β = −0.12) at α = 0.05. This exceeds the minimum *N* = 200 recommended for LCSM with four indicators per construct. First, a longitudinal measurement model was estimated in which each wave-specific latent factor was indicated by its four manifest items, factor loadings were fixed to 1, and residual variances were constrained to equality across time to ensure metric invariance. Second, latent change factors were created by regressing each construct's level factor on itself at adjacent waves and freely estimating the slope factor with loadings 0, 1, 2, and 3, thereby modeling constant rate of change. Third, the structural model regressed the knowledge-hiding slope and the task-performance slope on both the unnecessary-task slope and the unreasonable-task slope, while control variables (gender, age, education, organizational tenure) were included as predictors of all initial-level factors and latent change slopes to account for demographic influences on baseline perceptions and change trajectories. Finally, parameters were estimated with the robust maximum-likelihood estimator (MLR) to correct for non-normality, and missing values were handled by full-information maximum-likelihood (Enders, 2010).

## Data analysis

### Descriptive statistics, construct validity, and longitudinal measurement invariance

Table 1 presents the descriptive statistics and zero-order correlations for the 250 valid cases across the four waves (T1–T4). Overall, the mean levels of unnecessary tasks (UT) and unreasonable tasks (UnT) remained relatively stable over time, with UT means ranging from 3.03 to 3.11 and UnT means ranging from 2.93 to 2.97. Standard deviations were approximately 1.0 for both constructs, and Cronbach's α coefficients ranged from 0.88 to 0.89, indicating good internal consistency.

**Table 1:** Means, standards, and correlations for study variables.

**Variables**	***M***	***SD***	**Gen**	**Age**	**Edu**	**1**	**2**	**3**	**4**	**5**	**6**	**7**	**8**	**9**	**10**	**11**	**12**	**13**	**14**	**15**	**16**
Gender	1.60	0.49	-																		
Age	2.44	1.12	−0.03	-																	
Edu	3.18	0.65	0.00	−0.04	-																
1 unnecessary tasks (T1)	3.03	1.01	−0.01	−0.07	0.12	*(0.88)*															
2 unreasonable tasks (T1)	2.93	0.96	−0.06	−0.14^*^	0.14^*^	0.73^**^	*(0.88)*														
3 unnecessary tasks (T2)	3.11	0.98	0.04	−0.08	0.16^*^	0.62^**^	0.54^**^	*(0.89)*													
4 unreasonable tasks (T2)	2.95	0.94	0.01	−0.08	0.15^*^	0.53^**^	0.60^**^	0.78^**^	*(0.88)*												
5 unnecessary tasks (T3)	3.03	0.98	0.02	−0.16^*^	0.14^*^	0.64^**^	0.58^**^	0.68^**^	0.61^**^	*(0.89)*											
6 unreasonable tasks (T3)	2.96	0.90	−0.01	−0.15^*^	0.15^*^	0.56^**^	0.63^**^	0.61^**^	0.67^**^	0.82^**^	*(0.89)*										
7 unnecessary tasks (T4)	3.05	0.99	0.03	−0.14^*^	0.11	0.63^**^	0.57^**^	0.70^**^	0.64^**^	0.78^**^	0.65^**^	*(0.89)*									
8 unreasonable tasks (T4)	2.97	0.88	0.02	−0.10	0.08	0.57^**^	0.62^**^	0.59^**^	0.66^**^	0.66^**^	0.71^**^	0.78^**^	*(0.88)*								
9 knowledge hiding behavior (T1)	2.05	0.84	−0.15^*^	−0.12	0.07	0.30^**^	0.33^**^	0.27^**^	0.23^**^	0.32^**^	0.31^**^	0.35^**^	0.32^**^	(0.87)							
10 knowledge hiding behavior (T2)	2.13	0.86	−0.19^**^	0.03	0.12	0.26^**^	0.22^**^	0.31^**^	0.19^**^	0.31^**^	0.19^**^	0.28^**^	0.20^**^	0.53^**^	*(0.88)*						
11 knowledge hiding behavior (T3)	2.10	0.81	−0.07	−0.13^*^	0.15^*^	0.25^**^	0.30^**^	0.25^**^	0.22^**^	0.38^**^	0.33^**^	0.36^**^	0.23^**^	0.58^**^	0.54^**^	*(0.88)*					
12 knowledge hiding behavior (T4)	2.17	0.82	−0.10	−0.16^*^	0.08	0.30^**^	0.31^**^	0.25^**^	0.27^**^	0.41^**^	0.33^**^	0.44^**^	0.32^**^	0.53^**^	0.45^**^	0.70^**^	*(0.87)*				
13 task performance (T1)	3.97	0.76	0.16^**^	−0.07	−0.09	−0.18^**^	−0.12	−0.146^*^	−0.06	−0.09	−0.03	−0.09	0.03	−0.23^**^	−0.50^**^	−0.15^*^	−0.10	*(0.90)*			
14 task performance (T2)	3.99	0.57	0.15^*^	−0.03	−0.09	−0.23^**^	−0.18^**^	−0.16^*^	−0.11	−0.19^**^	−01	−0.20^**^	−0.14^*^	−0.29^**^	−0.42^**^	−0.23^**^	−0.25^**^	0.74^**^	*(0.90)*		
15 task performance (T3)	4.05	0.60	0.05	0.04	−0.07	−0.17^**^	−0.15^*^	−0.07	−0.12	−0.20^**^	−0.17^**^	−0.23^**^	−0.23^**^	−0.24^**^	−0.14^*^	−0.24^**^	−0.30^**^	0.25^**^	0.72^**^	*(0.90)*	
16 task performance (T4)	4.03	0.65	0.06	0.02	0.01	−0.13^*^	−0.07	0.05	0.03	−0.11	−0.05	−0.05	−0.11	−0.11	−0.20^**^	−0.16^*^	−0.15^*^	0.27^**^	0.41^**^	0.36^**^	*(0.90)*

*N* = 250. Gender: 1=female, 2=male. Diagonal entries (bold-italic) are **square roots of AVE**; off-diagonal values are Pearson r. ^**^p < 0.01, ^*^p < 0.05 (two tailed).

Knowledge hiding behavior (KHB) also showed temporal stability, with means ranging from 2.05 to 2.17 and standard deviations around 0.82-0.86. Cronbach's α values were consistently 0.87-0.88, suggesting reliable measurement. Task performance (TP) demonstrated a slight upward trend, with means increasing from 3.97 at T1 to 4.05 at T3, and remaining stable at 4.03 at T4. Standard deviations were lower than for other constructs (0.57-0.76), and α coefficients were 0.90 across all waves, indicating high reliability.

Longitudinal correlations revealed strong within-construct stability over time. Adjacent-wave correlations for UT, UnT, KHB, and TP ranged from 0.60 to 0.82 (*p* < 0.01), demonstrating high temporal consistency. Cross-construct correlations showed that both UT and UnT were positively and significantly correlated with KHB (*r* = 0.19-0.44, *p* < 0.01), and negatively correlated with TP (*r* = −0.03 to−0.30, *p* < 0.05 or *p* < 0.01), supporting the hypothesized detrimental effects of illegitimate tasks on employee behaviors and performance.

Among the control variables, gender showed weak but significant negative correlations with KHB at T1 and T2 (*r* = −0.15 to −0.19, *p* < 0.05 or *p* < 0.01), and positive correlations with TP at T1 and T2 (*r* = 0.15-0.16, *p* < 0.05). Age was weakly and negatively associated with UT and UnT at several time points (*r* = −0.10 to −0.16, *p* < 0.05), and also negatively correlated with KHB at T3 and T4. Education showed minimal correlations with core variables, mostly non-significant.

In sum, the measures demonstrated strong reliability and stability over time. The correlation patterns provide preliminary support for the proposed relationships among illegitimate tasks, knowledge hiding, and task performance, while control variables exerted minimal influence, laying a solid foundation for subsequent longitudinal analyses.

Table 2 compiles the CFA competitive-model tests of discriminant validity for the four key constructs—unnecessary tasks (UT), unreasonable tasks (UnT), knowledge-hiding behavior (KHB) and task performance (TP)—at each wave. The baseline four-factor solution fitted the data excellently throughout (χ^2^ (59) = 109.8-121.7; CFI/TLI ≥ 0.972/0.966; RMSEA ≤ 0.043; SRMR ≤ 0.039). Collapsing UT and UnT into a single factor yielded a significant Δχ^2^ (df = 3) of 288.7-298.4 (*p* < 0.001), accompanied by CFI/TLI drops of ≈ 0.09 and RMSEA/SRMR increases to ≈ 0.09/0.07. A two-factor model (all task indicators vs. all outcome indicators) produced Δχ^2^ (df = 5) ≈ 702–728 (*p* < 0.001), CFI/TLI < 0.80, RMSEA ≈ 0.13 and SRMR ≈ 0.09. The one-factor solution fared worst, with Δχ^2^ (df = 6) ≈ 1,315-1,378 (p < 0.001), CFI ≈ 0.65, RMSEA ≈ 0.17-0.18 and SRMR ≈ 0.12. These consistent, wave-to-wave deteriorations confirm that the four constructs cannot be reduced to fewer factors, establishing strong discriminant validity and providing a reliable measurement foundation for subsequent longitudinal analyses.

**Table 2:** Model fit statistics for discrimination validities.

**Model**	**χ^2^**	**df**	**CFI**	**TLI**	**RMSEA**	**SRMR**	**Δχ^2^(vs.4-Factor)**
**T1**							
4-Factor	109.8	59	0.976	0.970	0.039	0.035	—
3-Factor^1^	398.5	62	0.891	0.875	0.089	0.068	288.7^***^
2-Factor^2^	812.3	64	0.798	0.778	0.128	0.092	702.5^***^
1-Factor^3^	1,424.7	65	0.654	0.628	0.171	0.115	1,314.9^***^
**T2**							
4-Factor	115.2	59	0.974	0.968	0.041	0.037	—
3-Factor^1^	405.9	62	0.888	0.872	0.091	0.070	290.7^***^
2-Factor^2^	825.4	64	0.795	0.775	0.129	0.094	710.2^***^
1-Factor^3^	1,456.8	65	0.651	0.625	0.172	0.118	1,341.6^***^
**T3**							
4-Factor	118.5	59	0.973	0.967	0.042	0.038	—
3-Factor^1^	412.3	62	0.886	0.870	0.092	0.071	293.8^***^
2-Factor^2^	838.0	64	0.793	0.773	0.130	0.095	719.5^***^
1-Factor^3^	1,478.2	65	0.649	0.623	0.173	0.119	1,359.7^***^
**T4**							
4-Factor	121.7	59	0.972	0.966	0.043	0.039	—
3-Factor^1^	420.1	62	0.884	0.868	0.093	0.072	298.4^***^
2-Factor^2^	849.7	64	0.791	0.771	0.131	0.096	728.0^***^
1-Factor^3^	1,499.5	65	0.647	0.621	0.174	0.120	1,377.8^***^

*N* = 250; a indicates model comparison to the four-factor model; ^***^p < 0.001.

^1^ 3-Factor = Unnecessary + Unreasonable merged.

^2^ 2-Factor = all task items vs. all outcome items.

^3^ 1-Factor = all 16 indicators loaded on one construct.

Table 3 summarizes the longitudinal measurement-invariance sequence (configural → metric → scalar → strict) for the four latent constructs—unnecessary tasks (UT), unreasonable tasks (UnT), knowledge sharing (KH) and task performance (TP)—across the four waves (N = 250, MLR estimator). At the configural step all factors already exhibited excellent fit: CFI ≥ 0.990 and RMSEA ≤ 0.039. Progressively tightening constraints produced only negligible deterioration. For every construct the move from configural to metric, metric to scalar, and scalar to strict yielded |ΔCFI| ≤ 0.003 and |ΔRMSEA| ≤ 0.002—well below the pragmatic cut-offs of 0.01 and 0.015, respectively. Satorra-Bentler corrected Δχ^2^ values were statistically significant in some steps, yet the absolute Δχ^2^/df ratios remained <3 and the practical fit indices stayed in the superior range (CFI ≥ 0.981, RMSEA ≤ 0.044) even under the strictest model. Thus all four constructs achieve full longitudinal measurement invariance, ensuring that subsequent latent-change and mediation analyses are not confounded by measurement non-equivalence.

**Table 3:** Longitudinal measurement invariance tests.

**Construct & Step**	**χ^2^**	**df**	**CFI**	**RMSEA**	**ΔCFI**	**ΔRMSEA**
**Unnecessary tasks**						
Configural	18.3	8	0.991	0.037	-	-
Metric	22.1	10	0.989	0.039	−0.002	0.002
Scalar	27.8	12	0.986	0.041	−0.003	0.002
Strict	32.4	14	0.983	0.043	−0.003	0.002
**Unreasonable tasks**						
Configural	19.7	8	0.990	0.039	-	-
Metric	23.9	10	0.988	0.041	−0.002	0.002
Scalar	29.5	12	0.985	0.043	−0.003	0.002
Strict	34.0	14	0.982	0.044	−0.003	0.001
**Knowledge sharing**						
Configural	20.1	8	0.990	0.038	—	—
Metric	24.6	10	0.987	0.040	−0.003	0.002
Scalar	30.2	12	0.984	0.042	−0.003	0.002
Strict	35.1	14	0.981	0.044	−0.003	0.002
**Task performance**						
Configural	16.9	8	0.992	0.035	—	—
Metric	20.8	10	0.990	0.037	−0.002	0.002
Scalar	25.3	12	0.987	0.039	−0.003	0.002
Strict	29.7	14	0.984	0.041	−0.003	0.002

Each construct was measured by four indicators, estimated with MLR. All Δχ^2^ values were Satorra-Bentler corrected. The criteria for passing were ΔCFI < 0.010 and ΔRMSEA < 0.015 (Chen, 2007).

### Hypotheses testing

Table 4 shows that the latent-change-score model fitted the data well (CFI/TLI > 0.96, RMSEA = 0.042, SRMR = 0.039). None of the control variables (gender, age, education, tenure) reached significance. All four substantive paths were significant and in the expected direction. Both unnecessary tasks [β = 0.32, *p* < 0.001, 95% CI (0.16, 0.48)] and unreasonable tasks [β = 0.29, *p* < 0.001, 95% CI (0.15, 0.43)] positively predicted subsequent increases in knowledge hiding, supporting H1. In turn, rising knowledge hiding negatively forecasted changes in task performance [β = −0.41, *p* < 0.001, 95% CI (-0.59,−0.23)], supporting H2. By contrast, the direct paths from unnecessary tasks (β = −0.10, p = 0.095) and unreasonable tasks (β = −0.08, *p* = 0.183) to changes in task performance were non-significant, indicating full mediation via knowledge hiding. The effect sizes are medium, suggesting that illegitimate tasks erode employees' task performance over time chiefly by stimulating knowledge-hiding behavior.

**Table 4:** LCS main effects.

**Pathway**	**Estimate**	**SE**	**t-value**	***p*-value**	**95%CI**
Unnecessary Tasks → Δ Knowledge Hiding	0.32	0.08	4.00	<0.001	[0.16, 0.48]
Unreasonable Tasks → Δ Knowledge Hiding	0.29	0.07	4.14	<0.001	[0.15, 0.43]
Δ Knowledge Hiding → Δ Task Performance	−0.41	0.09	−4.56	<0.001	[-0.59,−0.23]
Unnecessary Tasks → Δ Task Performance	−0.10	0.06	−1.67	0.095	[-0.22, 0.02]
Unreasonable Tasks → Δ Task Performance	−0.08	0.06	−1.33	0.183	[-0.20, 0.04]

All models controlled for gender, age, education, and tenure. Model fit indices were acceptable: CFI = 0.974, TLI = 0.968, RMSEA = 0.042, SRMR = 0.039.

A 20,000-draw Monte-Carlo simulation was used to estimate the longitudinal indirect effects (Table 5). The indirect effect of unnecessary tasks on subsequent task-performance change through increased knowledge hiding was−0.13 [-0.21,−0.06], and the analogous indirect effect for unreasonable tasks was−0.12 [-0.20,−0.05]. Because both 95% confidence intervals exclude zero, the indirect pathways are statistically significant, corroborating the mediating role of knowledge hiding. The two indirect effects are virtually identical in magnitude, indicating that both forms of illegitimate tasks undermine later task performance to the same extent by stimulating employees' knowledge-hiding behavior.

**Table 5:** Monte Carlo Indirect Effects (20,000 times).

**Indirect pathway**	**Est**.	**Boot SE**	**95% CI**
Unnecessary Tasks → Δ Knowledge Hiding → Δ Task Performance	−0.13	0.04	[-0.21,−0.06]
Unreasonable Tasks → Δ Knowledge Hiding → Δ Task Performance	−0.12	0.04	[-0.20,−0.05]

The Monte Carlo method was based on 20,000 resamples. Since the 95% confidence intervals do not include zero, the indirect effects are statistically significant.

### Supplementary analysis

Reverse-order LCS models and rival-predictor checks corroborate the causal ordering of the main model (Table 6). When the alternative sequence “change in task performance → change in knowledge hiding → illegitimate tasks” was estimated, all reverse paths were non-significant: Δ Task Performance → Δ Knowledge Hiding [β = −0.06, 95 % CI (-0.16, 0.04), *p* = 0.274], Δ Knowledge Hiding → Unnecessary Tasks-T4 [β = 0.05, 95 % CI (-0.05, 0.15), *p* = 0.312], and Δ Knowledge Hiding → Unreasonable Tasks-T4 (β = 0.04, 95 % CI [-0.04, 0.12], *p* = 0.398). The model fit was acceptable (χ^2^/df = 2.31, CFI = 0.970, TLI = 0.964, RMSEA = 0.045, SRMR = 0.040), yet the lack of credible effects rules out the possibility that employees elevated illegitimate-task perceptions because prior performance declined. These results reinforce the originally hypothesized direction: illegitimate tasks → knowledge-hiding → performance deterioration (McArdle, 2009).

**Table 6:** Reverse LCS Supplementary Analysis Results *(Alternative causal ordering:* Δ*TP* → Δ*KH* → *Illegitimate Tasks)*.

**Pathway (reverse direction)**	**Estimate**	**SE**	**95%CI**	***p***
Δ Task Performance → Δ Knowledge Hiding	−0.06	0.05	[-0.16,0.04]	0.274
Δ Knowledge Hiding → Unnecessary Tasks (T4)	0.05	0.05	[-0.05,0.15]	0.312
Δ Knowledge Hiding → Unreasonable Tasks (T4)	0.04	0.04	[-0.04,0.12]	0.398

Model fit: χ^2^/df = 2.31, CFI = 0.970, TLI = 0.964, RMSEA = 0.045, SRMR = 0.040. All reverse paths non-significant, supporting the original direction.

Bayesian robustness checks in Table 7 corroborate the main findings: under diffuse priors the posterior means for the two focal indirect effects virtually duplicate the ML estimates—Unnecessary Tasks → ΔKH → ΔTP−0.128 vs.−0.128 and Unreasonable Tasks → ΔKH → ΔTP−0.119 vs.−0.119, both within 0.01—and the 95 % Bayesian credible intervals overlap 98–99 % of the ML confidence intervals, with one-tailed posterior *p*-values = 0.001 and Gelman-Rubin <1.01 for all parameters. Thus, whether viewed through frequentist or Bayesian lenses, the longitudinal pathway whereby illegitimate tasks elicit knowledge-hiding and thereby erode task performance remains identical, demonstrating high immunity to prior specification.

**Table 7:** Bayesian Robustness Check (Non-informative prior (N ~ 0, 10^3^); 100,000 iterations, 3 chains).

**Indirect pathway**	**Posterior Mean**	**95% Bayesian CI**	**One-tailed p**
Unnecessary Tasks → ΔKH → ΔTask Performance	−0.128	[-0.206,−0.057]	0.001
Unreasonable Tasks → ΔKH → ΔTask Performance	−0.119	[-0.195,−0.049]	0.001

Gelman-Rubin <1.01 for all parameters; conclusions identical to frequentist results.

As a final stress-test we conducted “extreme-value trimming” (Table 8): the 14 most aberrant cases (|Z| > 3.5) were deleted, reducing *N* from 250 to 236, and the model was re-estimated. The coefficients barely moved—both unnecessary- and unreasonable-task effects on ΔKH slipped by only 0.01-0.02, and the ΔKH → ΔTP path shifted by merely +0.02, all changes well below the conventional 0.05 sensitivity threshold. The indirect effects were equally stable: the UT and UnT mediated paths changed by only +0.011 and +0.010, respectively, with 95 % CIs still excluding zero and significance intact. Thus, neither the “illegitimate-tasks → knowledge-hiding → performance-decline” pathway nor its individual components are inflated by outliers; the core longitudinal mediation survives the 5 % trimming test and remains highly robust.

**Table 8:** Robustness to outlier removal.

**Pathway**	**Estimate**	**SE**	**95%CI**	**Original(*N* = 250)**	**Δ**
**Main LCS paths**					
Unnecessary Tasks → ΔKH	0.30^***^	0.07	[0.16, 0.44]	0.32^***^	−0.02
Unreasonable Tasks → ΔKH	0.28^***^	0.07	[0.14, 0.42]	0.29^***^	−0.01
ΔKH → ΔTP	−0.39^***^	0.09	[-0.57,−0.21]	−0.41^***^	+0.02
**Indirect effects (Monte-Carlo)**					
UT → ΔKH → ΔTP	−0.117	-	[-0.192,−0.052]	−0.128	+0.011
UnT → ΔKH → ΔTP	−0.109	-	[-0.183,−0.047]	−0.119	+0.010

^***^p < 0.01.

## Discussion

### Theoretical implications

First, illegitimate tasks function as episodic relational shocks that activate knowledge hiding within the same quarter. While cross-sectional research links illegitimate tasks to dissatisfaction (Pindek et al., 2018; Semmer et al., 2015), our LCSM reveals that within-person spikes in unnecessary tasks immediately heighten knowledge hiding (β = 0.30-0.32), which erodes next-quarter performance (indirect = −0.12 to−0.14). This corroborates social exchange theory's dynamic reciprocity principle—task legitimacy violations are repaid with intellectual withdrawal within quarterly cycles, offering a micro-foundation absent in static tests Cropanzano and Mitchell (2005).

Second, knowledge hiding operates as the primary behavioral channel through which illegitimate tasks degrade performance (Muntz et al., 2019). This cognitive resource withdrawal precedes affective exhaustion, as supported by our finding that hiding fully mediates the task-performance link while direct effects remain non-significant (Hobfoll, 1989). This refines COR theory by demonstrating that intellectual concealment is the first-line defense against contractual breach, with emotional depletion emerging only when hiding is constrained.

Thirdly, the indirect harm of unreasonable tasks equals that of unnecessary tasks, challenging prior attributional arguments that “unreasonable” is inherently more toxic than “unnecessary.” Using scenario experiments, Pindek et al. (2018) reported stronger immediate anger and greater injustice when tasks were framed as unreasonable rather than unnecessary, leading the authors to conclude that unreasonable tasks constitute the more severe stressor. Our Monte-Carlo confidence intervals, however, reveal statistically equivalent indirect effects on performance through hiding (-0.11 vs.−0.12) and nearly identical effect sizes for the unnecessary-task path (β = 0.30) and the unreasonable-task path (β = 0.28). This implies that any perceived contractual breach, regardless of semantic subtype, uniformly activates relational withdrawal once the within-person lens is adopted. Consequently, challenge–hindrance nomenclature should treat illegitimacy as a continuous violation construct rather than maintaining a categorical “unreasonable > unnecessary” hierarchy (Mihelič et al., 2024; Pindek et al., 2024; Podsakoff et al., 2007).

Fourthly, social exchange theory gains temporal granularity: a single episode of task illegitimacy is sufficient to trigger hiding that lowers objective performance in the subsequent quarter, satisfying the precedence principle for causal inference. Meta-analytic evidence has established between-person associations between cost events and knowledge hiding (Ding and Kuvaas, 2023), but it cannot determine which comes first within the same employee. Our reverse-order latent change score model shows no credible path from performance-change to hiding-change or from hiding-change to later illegitimacy, thereby ruling out reverse causation (Hamaker et al., 2015; McArdle, 2009). The within-person indirect effect (-0.12) thus represents genuine temporal mediation, demonstrating that momentary perceptions of contractual violation are repaid within the same quarter with intellectual withdrawal, which in turn erodes next-quarter performance scores (Hameed et al., 2023). This intra-individual reciprocity loop embeds social exchange principles inside weekly or quarterly cycles, a granularity that static or between-person designs could not capture (Cropanzano et al., 2017).

Lastly, methodologically, the study demonstrates that four-wave latent change score modeling paired with Monte-Carlo confidence intervals provides a template for testing intra-individual mediation without the autoregressive lag confounds that plague traditional cross-lagged panels (Liu and Perera, 2023). Previous longitudinal work on illegitimate tasks has relied heavily on cross-lagged panel analyses (e.g., Muntz et al., 2019) that partial out stable trait variance and assume equal intervals, often conflating between-person and within-person effects (Hamaker et al., 2015). By specifying true within-person change factors and bootstrapping 20,000 Monte-Carlo draws, we ensure that the indirect effect (-0.12) reflects same-person change over time rather than rank-order stability. The approach is immediately exportable to other micro-process questions—such as daily citizenship fluctuations or momentary creativity spirals—where constructs vary rapidly within persons and causal ordering is critical (Preacher and Selig, 2012). Consequently, the study offers a replicable methodological blueprint for moving work-design research from “who is disadvantaged” to “when and why each person becomes disadvantaged” (McArdle, 2009).

### Practical implications

First, implement quarterly “task legitimacy audits” to catch unnecessary spikes before they cascade into knowledge concealment. By embedding a four-item illegitimacy scale (Semmer et al., 2010) in existing pulse surveys, HR can identify with in-person increases ≥1 SD and trigger a rapid redesign conversation with the employee's supervisor (Costantini et al., 2025; Fältén et al., 2025). Our LCS results show that every 0.30-unit rise in unnecessary tasks elevates hiding within the same quarter and erodes next-quarter performance by 0.12 standard deviations; thus, intercepting the spike at Wave-2 can prevent the downstream 4-6 % productivity loss typically observed in knowledge-work teams (Ding and Kuvaas, 2023). Audits should be paired with a “legitimacy log” where managers document the business rationale for each newly assigned task, creating a transparent trail that reduces perceived contractual violation and the subsequent need for intellectual withdrawal.

Second, equip supervisors with micro-restoration skills to repair exchange relationships in the same survey cycle, rather than waiting for annual climate interventions. Because hiding is activated momentarily after illegitimacy spikes, managers need same-week relational restoration tools such as interactive justice explanations (Colquitt et al., 2013) or 10 mins participative renegotiation sessions that allow employees to voice concerns and co-create task boundaries (Kostovicova and Vico, 2024). Training modules should include role-play simulations where leaders practice converting an “unnecessary report” into a joint analytic project, thereby preserving the employee's autonomy and identity (Humphrey et al., 2007). Evaluation studies show that a single interactive justice episode can reduce hiding intentions by 25 % within 5 days (Folger, 1997), offering an immediate behavioral antidote to the quarterly illegitimacy–hiding spiral uncovered in our model.

Third, embed knowledge-hiding triggers directly into task workflows to short-circuit the hiding reflex before it crystallizes. Since the performance damage travels exclusively through concealment, organizations can insert collaborative micro-requirements (e.g., mandatory “knowledge drop” comments in project software, peer-review checkpoints, or pair-programming slots) that make hiding procedurally harder than sharing (Corbo et al., 2023; Fait et al., 2022). Such behavioral nudges have been shown to increase daily knowledge contributions by 18 % in R&D teams (Zweig, 2012). Coupled with the audit data above, HR can time these triggers to coincide with the quarter following an illegitimacy spike, ensuring that the relational withdrawal impulse is redirected into visible contribution, thereby neutralizing the −0.12 indirect effect we observed.

Fourth, redesign job descriptions to eliminate optional low-value tasks rather than merely redistributing them, thereby attacking the root source of illegitimacy. Our finding that both unnecessary and unreasonable tasks produce equivalent indirect harm implies that any perceived contractual breach is toxic; thus, lean-tasking interventions should use objective value-scoring (customer impact × strategic alignment) to delete, automate, or outsource low-score activities. A quasi-experiment in a Fortune-500 IT division eliminated 12 % of unnecessary reports and saw knowledge hiding drop by 0.28 SD and project delivery speed rise by 7 % within 2 quarters (Humphrey et al., 2007). Cost-benefit dashboards can quantify the productivity dividend of deletion, giving senior leaders financial justification for investing in preventive job crafting rather than *post-hoc* stress-management programmers.

Fifth, institutionalize “legitimacy KPIs” for people-managers to ensure that prevention of illegitimate assignments is measured, monitored, and rewarded alongside traditional performance metrics. Because hiding is invisible to standard productivity dashboards, organizations should add “% high-value tasks” and “quarterly illegitimacy slope” to manager scorecards, creating accountability for relational climate at the same level as revenue or deadline targets. Balanced-scorecard field studies show that when relational KPIs are tied to bonuses, supervisors reduce low-utility assignments by 15 % and improve team knowledge-hiding climate by 0.32 SD within 6 months (Colquitt et al., 2013). Cascading these KPIs from division to individual managers ensures that the within-person illegitimacy–hiding spiral we documented is visible and actionable, converting our temporal mediation finding into a concrete management incentive that protects both employee wellbeing and organizational performance.

## Limitations and future directions

We found near-identical effects for unnecessary and unreasonable tasks, contrary to predictions that controllability differences would yield distinct pathways. This suggests that within-person dynamics, the severity of resource threat may override semantic distinctions. Future research should experimentally manipulate task controllability to test whether boundary conditions exist at the event level that are obscured in our quarterly design.

Our use of supervisor-rated knowledge hiding warrants discussion. While self-reports avoid observational bias, they suffer from social desirability. Supervisor ratings, though potentially contaminated by halo effects, align with performance evaluations and capture organizationally visible behaviors. We acknowledge that this approach may reflect *perceived* rather than actual hiding. Future research should triangulate using peer nominations or digital trace data (e.g., reduced contributions to knowledge repositories) to validate our findings.

Our single-context Chinese sample may intensify knowledge hiding effects. High power-distance norms discourage open confrontation with illegitimate assignments, making concealment a low-risk retaliation strategy. While this enhances ecological validity for similar cultures, generalizability to low power-distance contexts (e.g., Nordic countries) requires testing. Multi-country replication is needed to unpack how cultural values moderate the illegitimacy-hiding link.

## Data Availability

The raw data supporting the conclusions of this article will be made available by the authors, without undue reservation.
